# Hybrid phenylthiazole and 1,3,5-triazine target cytosolic leucyl-tRNA synthetase for antifungal action as revealed by molecular docking studies

**DOI:** 10.1186/2193-9616-1-3

**Published:** 2013-02-12

**Authors:** Udaya Pratap Singh, Hans Raj Bhat, Prashant Gahtori, Ramendra K Singh

**Affiliations:** 1 Department of Pharmaceutical Sciences, Sam Higginbottom Institute of Agriculture, Technology & Sciences, Deemed University, Allahabad, 211007 India; 2 Faculty of Pharmacy, Uttarakhand Technical University, Deharadun, 248007 India; 3 Nucleic Acids Research Laboratory, Department of Chemistry, University of Allahabad, Allahabad, 211002 India; 4 Archimedes DoRa5 Visiting Fellow, Institute of Chemistry, Division of Bio-organic Chemistry, Institute of Chemistry, University of Tartu, Tartu, Estonia

**Keywords:** Leucyl-tRNA synthetase, Docking, phenylthiazole, 1,3,5-triazine

## Abstract

**Background:**

Leucyl-tRNA synthetase (LeuRS) is one of the essential enzymes belonging to the family of aminoacyl-tRNA synthetases (aaRSs), which executes the translation of genetic code by catalyzing the specific attachment of amino acids to their cognate tRNAs. This process is very crucial for survival of micro-organism and thus the inhibition of LeuRS offered a novel and lucrative target for developing new antimicrobials.

**Findings:**

Docking studies using hybrid phenylthiazole-1,3,5-triazine derivatives revealed that these molecules acted as probable inhibitors of candida albicans cytosolic leucyl-tRNA synthetase

**Conclusion:**

The conjugates of phenylthiazole and 1,3,5-triazine can act as lead molecules towards the development of potential leucyl-tRNA synthetase inhibitors on the basis of molecular docking runs, which contribute to the possible mechanism of antifungal activity of these analogues.

## Introduction

Infections caused by opportunistic pathogenic fungi, especially candida species, *cryptococcus neoformans* and *aspergillus fumigates* are associated with high morbidity and mortality in immunocompromised patients (Pfaller and Diekema, 2004). Additionally, the dramatic rise in the prevalence of fungal resistance, currently poses a serious threat to public health worldwide (Ghannoum and Rice, 1999;Pfaller 2012). However, combined with improvements in performances, standardization of antifungal susceptibility testing and new drug discovery have drawn attention to the problem of antifungal resistance (Vanden Bossche et al., 1998).

**Figure 1:**
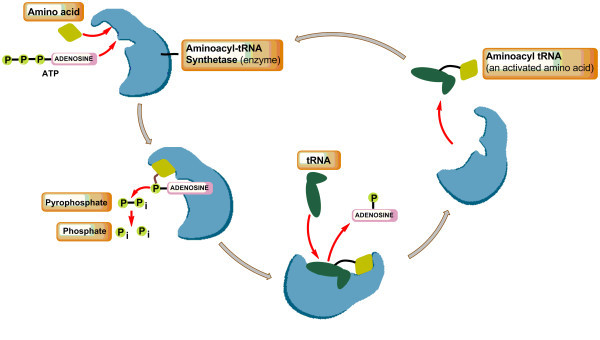
**Role of aminoacyl-tRNA synthetase (aaRS) enzyme in translating the genetic code by catalyzing the specific attachment of amino acids to their cognate tRNAs.**

Aminoacyl-tRNA synthetase (aaRS) enzymes have recently gained focus of attention as novel potential target for antimicrobial drug research (Pohlmann and Brötz-Oesterhelt, 2004). They perform a crucial role in translating the genetic code by catalyzing the specific attachment of amino acids to their cognate tRNAs in a two step reaction: activation of amino acid with ATP to form enzyme-bound aminoacyl-AMP (with release of pyrophosphate), and transfer of amino acid moiety to cognate tRNA, releasing AMP and charged tRNA (Figure 1). Based on the architecture of their catalytic domains, aaRSs belong to two distinct classes. Class I enzymes contain a typical Rossman fold in the active site, with the HIGH and KMSKS motifs that stabilize the transition state of the reaction for amino acid activation using ATP-binding energy. Class II enzymes harbor an antiparallel β-sheet domain that provides a rigid template for amino acid and ATP binding, with three characteristic motifs required for dimerization and substrate binding (Hurdle et al. 2005). A newly discovered antifungal agent AN2690, (under clinical investigation) reported to act by inactivating fungal LeuRS, a class I aaRS enzyme is responsible for charging leucine to its cognate tRNA correctly (Kim et al. 2003). As a result, design and discovery of LeuRS inhibitors surfaces as a prolific approach to attenuate microorganism growth for exploring novel antifungal agents via arrest of fungal protein synthesis (Hendrickson et al. Hendrickson and Schimmel 2003; Rock et al., 2007).

Molecular docking is widely accepted and frequently used tool in systems biology and computer-assisted drug discovery. It is used to predict the preferred orientation, affinity and activity of ligands (small molecules) into the active site of target protein through a process involving series of steps. In continuation of our research endeavour on discovery of novel anti-infective agents from 1,3,5-triazines (Gahtori et al. 2009; Singh et al. 2011; Bhat et al. 2011; Gahtori et al. 2012a; Gahtori et al. 2012b; Gahtori and Ghosh et al. 2012; Ghosh et al. 2012; Bhat et al. 2012, Singh et al. 2012). We have tried to work out the inhibitory effect of hybrid phenylthiazole-1,3,5-triazines on the *candida albicans* cytosolic leucyl-tRNA synthetase editing domain through molecular docking studies to elucidate probable mechanism of action of 1,3,5-triazine as antifungal agent.

## Experimental

### Molecular docking studies

The 3D X-ray crystal structure of benzoxaborole-AMP adduct docked into the *candida albicans* cytosolic leucyl-tRNA synthetase editing domain was used as a target protein model for this study (2wfg.pdb). All computational analyses were carried out using Discovery Studio 2.5 (DS 2.5, Accelrys Software Inc., San Diego; http://www.accelrys.com).

### Preparation of receptor

The target protein complexed with benzoxaborole-AMP adduct was taken, the ligand benzoxaborole-AMP adduct extracted, and the bond orders were corrected. The hydrogen atoms were added, their positions optimized using the all-atom CHARMm (version c32b1) forcefield with Adopted Basis set Newton Raphson (ABNR) minimization algorithm until the root mean square (r.m.s) gradient for potential energy was less than 0.05 kcal/mol/Å (Brooks et al. 1983; Momany and Rone 1992). Using the 'Binding Site' tool panel available in DS 2.5, the minimized *candida albicans* cytosolic leucyl-tRNA synthetase editing domain was defined as receptor, and binding site was defined as volume occupied by the ligand in the receptor, with a radius of 5 Å. The centre of the sphere was taken to be the centre of the binding site shape, and side chains of the residues in the binding site within the radius of the sphere were assumed to be flexible during refinement of post-docking poses.

### Ligand setup

Using the built-and-edit module of DS 2.5, the hybrid molecules of phenylthiazole and 1,3,5-triazine were built, all-atoms CHARMm forcefield parameterization assigned and then minimized using the ABNR method. A conformational search of the ligand was carried out using a stimulated annealing molecular dynamics (MD) approach. These ligands were heated to a temperature of 700 K and then annealed to 200 K. Thirty such cycles were carried out. The transformation obtained at the end of each cycle was further subjected to local energy minimization, using the ABNR method. The 30 energy-minimized structures were then superimposed and the lowest energy conformation occurring in the major cluster was taken to be the most probable conformation.

### Docking and scoring

LigandFit (Venkatachalam et al. 2003) protocol of DS 2.5 was used for the docking of ligands with *candida albicans* cytosolic leucyl-tRNA synthetase editing domain. The LigandFit docking algorithm combines a shape comparison filter with a Monte Carlo conformational search to generate docked poses consistent with the binding site shape. These initial poses are further refined by rigid body minimization of the ligand with respect to the grid based calculated interaction energy using the Dreiding forcefield (Mayo et al. 1990). The receptor protein conformation was kept fixed during docking, and the docked poses were further minimized using all-atom CHARMm (version c32b1) forcefield and smart minimization method (steepest descent followed by conjugate gradient) until r.m.s gradient for potential energy was less than 0.05 kcal/mol/Å. The atoms of ligand and the side chains of the residues of the receptor within 5 Å from the centre of the binding site were kept flexible during minimization. Finally, binding affinity of ligand towards the target protein were determined on the basis of scoring parameter PLP1 (Gehlhaar et al. 1995), PLP2 (Gehlhaar et al. 1999), PMF (Muegge and Martin 1999), Lig Internal Energy and Dock Score in post-docked pose of ligand. Results are presented in Tables 1 and 2.

**Table 1:** **Interaction generated between ligand-receptor after docking**

Sample	H-bonding				Pi-cation monitor			
	***Bond***	Distance*	***Donor atom***	***Acceptor atom***	***Bond***	Distance*	***End1***	***End2***
**1**	1:H30 –ASP422:OD1	2.34	H30	OD1	1 - Lys483:NZ	4.24	1	NZ
					1 - Lys483:NZ	5.40	1	NZ
**2**	Lys483:HZ3: 2:N14	2.45	HZ3	N14	1 - Lys483:NZ	4.78	2	NZ
	2: H30 – ASP422:OD1	2.30	H30	OD1	1 - Lys483:NZ	4.30	2	NZ
**3**	3:H32 – ASP422	2.09	H32	OD1	3 – Lys 483:NZ	4.23	3	NZ
					3 - Lys407:NZ	5.01	3	NZ
					3 - Lys483:NZ	5.36	3	NZ
**4**	LYS407:HZ2 - N3: 4	2.03	HZ2	N3	4 - LYS483:NZ	3.46	4	NZ
	LYS483:HZ3 - N5: 4	2.42	HZ3	N5				
	4:H32 - ASP421:OD2	1.28	H32	H32				
**5**	NO			NO	5 -Lys483:NZ	3.46	5	NZ
**6**	LYS483:HZ1 – N1	1.87	HZ1	N1	6 - LYS483:NZ	3.16	6	NZ
	LYS483:HZ1 - N14	2.08	HZ1	N14	6 - LYS407:NZ	3.71	6	NZ
	LYS483:HZ2 – N14	2.28	HZ2	N14	6 - LYS483:NZ	4.61	6	NZ
**7**	LYS407:HZ1- N5:7	2.40	HZ1	N5	7- LYS483:NZ	3.72	7	NZ
	LYS407:HZ2 – N14:7	2.35	HZ2	N14	7 - LYS407:NZ	3.75	7	NZ
	LYS407:HZ3 – N5:7	2.21	HZ3	N5	7 - LYS483:NZ	4.93	7	NZ
**8**	NO				8 - LYS483:NZ	3.19	8	NZ
					8 - LYS407:NZ	3.94	8	NZ
					8 - LYS483:NZ	4.46	8	NZ
					8 - LYS407:NZ	5.40	8	NZ
					8 - LYS407:NZ	4.91	8	NZ
**9**	LEU317:HN – O47:9	1.89	HN	O47	9 - LYS407:NZ	3.13	9	NZ
	LYS407:HZ1 –N1:9	2.40	HZ1	N1	9 - LYS407:NZ	3.00	9	NZ
	LYS407:HZ2 – N14:9	1.61	HZ2	N14	9 - LYS483:NZ	3.55	9	NZ
	LYS407:HZ3 – N1:9	1.99	HZ3	N1				
**10**	LYS407:HZ2 – N14:10	2.089	HZ2	N14	10 - LYS483:NZ	3.43	10	NZ
	LYS483:HZ1 –N5:10	2.276	HZ1	N5	10 - LYS407:NZ	4.42	10	NZ
					10 - LYS483:NZ	5.29	10	NZ
**11**	LYS407:HZ2 - N14: 11	1.97	HZ2	N14	11 - LYS483:NZ	3.44	11	NZ
	LYS483:HZ1 - N5: 11	2.00	HZ1	N5	11 - LYS483:NZ	4.09	11	NZ
	11 :H41 - TYR487:OH	1.78	H41	OH	11 - ARG318:NE	5.74	11	NE
					11 - LYS407:NZ	3.72	11	NZ
					11- LYS407:NZ	4.75	11	NZ
**12**	ARG318:HH11 - O41: 12	1.79	HH11	O34	12 - LYS483:NZ	4.16	12	NZ
	LYS407:HZ2 - N14: 12	2.39	HZ2	N14	12 - LYS483:NZ	4.48	12	NZ
	SER476:HG -O34: 12	1.63	HG	O34	12 - LYS407:NZ	3.93	12	NZ
					12 - LYS483:NZ	5.99	12	NZ
**13**	13 :N7 - ALA315:O	3.18	N7	ALA315:O	13 - LYS483:NZ	4.18		
	13:N8 - TYR487:OH	2.60	N8	TYR487:OH	13 - LYS407:NZ	4.23		
					13 - LYS407:NZ	3.82		
**14**	LYS483:HZ1 – N5: 14	2.36	HZ1	N5	14 - LYS483:NZ	3.73	14	NZ
	14:N8 - TYR487:OH	2.56	N8	OH	14 - LYS407:NZ	4.24	14	NZ
					14 - LYS407:NZ	3.50	14	NZ
**15**	LYS407:HZ2 - N14: 15	2.88	HZ2	N14	15 - LYS483:NZ	3.33	15	NZ
	SER476:HG -O37: 15	1.91	HG	O37	15 - LYS407:NZ	5.18	15	NZ
	LYS483:HZ1 - N5: 15	2.20	HZ1	N5				
	TYR487:HH - N3: 15	2.44	HH	N3				

*: Distance in Å.

**Table 2:** **Scoring profile of ligands**

Name	-PLP1	-PLP2	-PMF	Lig-internal_energy	Dock score
**1**	82.49	61.74	62.31	-3.95	63.31
**2**	68.04	60.34	106.12	-5.50	61.94
**3**	78.68	67.35	106.87	-3.75	60.99
**4**	72.98	66.45	86.17	-3.31	63.77
**5**	71.74	68.03	91.95	-4.38	67.40
**6**	78.73	62.82	99.99	-4.79	60.00
**7**	45.01	47.38	42.44	-10.55	67.62
**8**	125.57	126.27	153.2	-10.20	61.60
**9**	42.84	43.3	93.07	-6.62	50.58
**10**	89.98	86.07	126.84	-7.76	67.88
**11**	97.75	94.22	128.93	-6.95	74.25
**12**	93.01	82.13	144.09	-1.94	56.43
**13**	101.68	85.74	121.12	-7.60	73.01
**14**	106.93	94.21	117.26	-7.82	75.68
**15**	85.8	80.86	117.03	-7.44	74.28

## Results and discussion

### Docking

**Figure 2:**
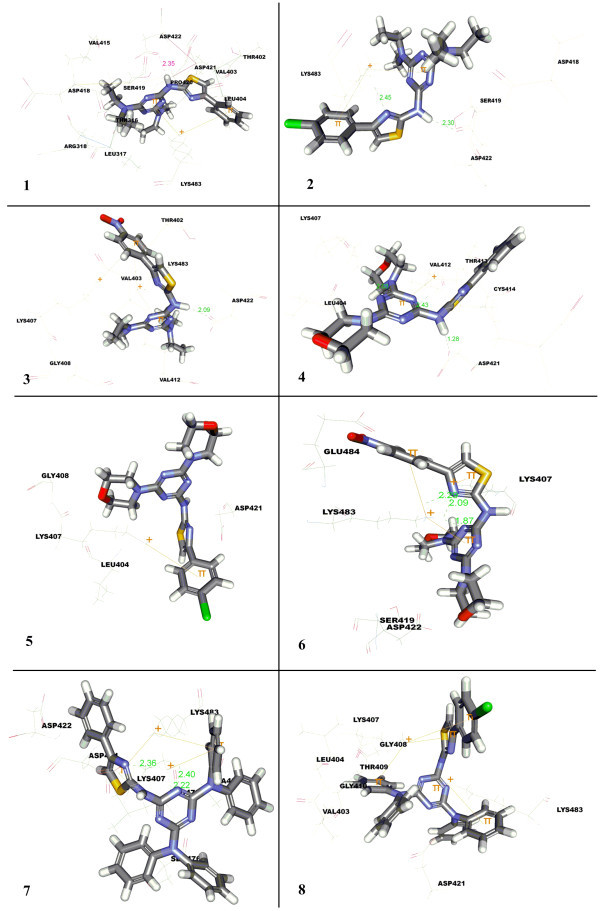
**Docked pose of compounds 1-8 in Leu-tRNA synthetase.**

**Figure 3:**
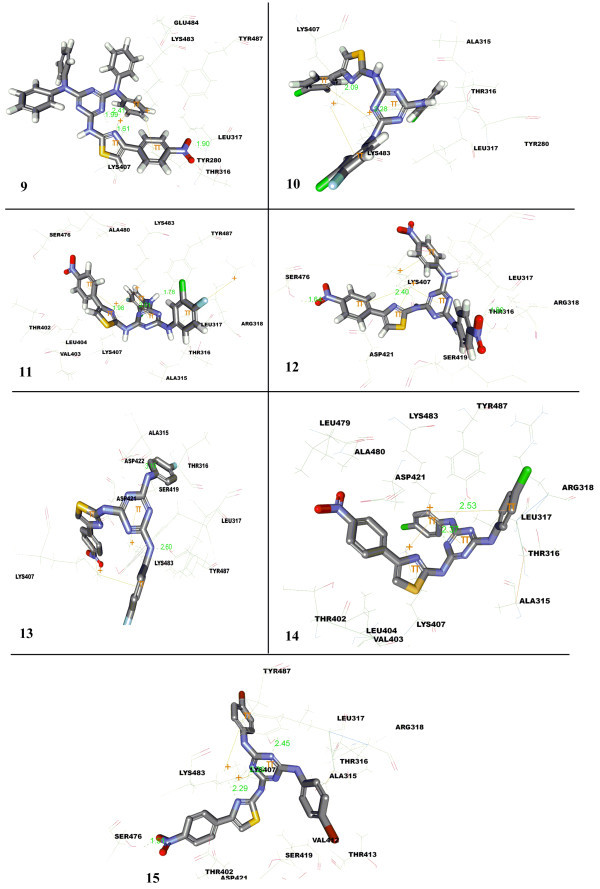
**Docked pose of compounds 9-15 in Leu-tRNA synthetase.**

Till date a very few leucyl-tRNA synthetase inhibitors have been reported as antifungal agents. One such molecule AN2690, a benzoxaborole derivative as potent non-competitive inhibitor is under development. In the present study, molecular docking studies of hybrid phenylthiazole-1,3,5-triazine derivatives have been carried onto the binding site of *candida albicans* cytosolic leucyl-tRNA synthetase editing domain to exemplify the orientation & binding affinity, and Dockscores calculated from the docked conformations of the thymidylate synthetase -inhibitor complexes using LigandFit within DS 2.5. Docking results were discussed on the parameters such as hydrogen bond, pi-pi(hydrophobic) and non-polar pi-cation (non-covalent) interactions. According to Gallivan and Dougherty, pi-cation interaction energies are considered of the same order of magnitude as hydrogen bonds or salt bridges and play an important role in molecular recognition and interaction with ligands. These interactions of the title compounds have been discussed, if the interatomic distance fell below 6 Å (Gallivan and Dougherty 1999). Considering the magnitude of interacting forces for receptor-ligand interaction, the entire set of molecules were rigorously docked onto the active site of thymidylate synthetase, using the same protocol and analyzed through above mentioned parameters and results presented in Figure 2 (compounds 1-8) and Figure 3 (compounds 9-15), Tables 1 and 2.

It was revealed that entire molecules interacted in similar fashion with thymidylate synthetase by making use of hydrogen bonding and pi-cation interaction with considerable affinity, albeit, no pi-pi interactions were observed. From Table 1, two pi-cation interactions have been reported with compound **1** having iso-propyl amine connected to triazine and Lys483 involving phenyl of thiazole and 1,3,5-triazine with a distance of 4.24 Å and 5.24 Å, respectively. Additionally, formation of one hydrogen bond was also reported through hydrogen of amine connecting thiazole to 1,3,5-triazine. Introduction of 4-chloro at the phenyl connected to thiazole created an additional H-bond between nitrogen of thiazole and Lys483, as observed with compound **2**. On replacing 4-chloro with 4-NO_2_, compound **3** the additional H-bond created earlier was lost and new pi-cation interactions were observed between phenyl of thiazole and Lys407. Further, di-substitution of 1,3,5-triazine with morpholine fragment in compound **4** (intact phenyl on thiazole) yielded three hydrogen bonds, two using 3^rd^ and 5^th^ nitrogen of triazine with Lys407 (2.03 Å) and Lys483 (2.53 Å), and third using hydrogen connecting thiazole to triazine with Lys483(3.46 Å). Surprisingly, no hydrogen bond was reported in the case of compound **5** but for a single pi-cation interaction with 1,3,5-triazine and Lys483 (3.46 Å). Presence of 4-NO_2_ at phenyl of thiazole, compound **6**, led to the generation of three hydrogen bonds with Lys483, two with nitrogen of thaizole (2.08 and 2.28 Å) and last H-bond with nitrogen of triazine (1.87 Å). Three pi-cation interactions were also reported in the case of compound **6,** of which two with Lys 483 using 1,3,5-triazine and phenyl of thiazole with effective distance of 3.16 and 4.61 Å, respectively and the last using thiazole with Lys407 (3.71 Å). Three hydrogen and pi-cation interactions were displayed by compound **7** having diphenyl amine attached to both wings of 1,3,5-traizine. Lys 407 is the only amino acid responsible for generation of all three hydrogen bonds, two with nitrogen of 1,3,5-triazine (2.40 Å and 2.21 Å) and the last hydrogen bond through nitrogen present in thiazole (2.35 Å). While, two pi-cation interactions were observed through the involvement of one of the phenyl ring connected to 1,3,5-triazine with Lys407 (3.75 Å) and Lys483 (4.93 Å) and other by thiazole with Lys483 (3.72 Å). Markedly, no hydrogen bond was revealed by compound **8**, (introduction of 4-cholro in phenyl of thiazole), albeit reported formation of five pi-cation bonds with Lys407 (with thiazole, 3.94 Å; phenyl of 1,3,5-triazine, 5.40 Å and phenyl of thiazole, 4.91 Å) and Lys483 (with 1,3,5-triazine, 3.19 Å and phenyl of 1,3,5-triazine 4.46 Å) were observed. The H-bond formation with protein target was restored on replacing 4-Cl with 4-NO_2_ resulting in the formation of additional hydrogen bonds by Leu317 with oxygen of 4-NO_2_ on phenyl of thiazole and four by Lys407 with nitrogen of 1,3,5-triazine (1.99 Å, 2.04 Å) and nitrogen of thiazole (1.61 A), as observed in compound **9**. Introduction of di-halogen substituted phenyl amine on both the faces of 1,3,5-triazine (**10**), led to creation of two hydrogen bonds (nitrogen of thiazole with Lys407, 2.08 Å and 5^th^ nitrogen of 1,3,5-triazine) and three pi-cation interactions of which two by Lys483 with 1,3,5-triazine (3.43 Å) and phenyl of thiazole (5.29 Å), and the last one through di-halogen substituted phenyl amine of 1,3,5-triazine amid Lys407. Insertion of 4-NO_2_ in the place of 4-Cl on phenyl thiazole ring (**11**) led to an additional H-bond formation by Tyr487 (OH) through amine bridge connecting 1,3,5-triazine and pi-cation through Arg318 with phenyl of 1,3,5-triazine (5.74 Å). Amino acid residues, Arg315 and Ser476 were responsible for creation of two H- bonds with compound **12**, bearing 4-NO_2_ on phenyl amine ring connected to 1,3,5-triazine system (1.79 Å and 1.63 Å respectively). The amine bridge connecting distant 4-fluoro phenyl amine to 1,3,5-triazine (**13**) resulted in the formation of two hydrogen bonds will Ala315 (2.60 Å) and Tyr457 (3.68 Å). Furthermore, the compound **13** also exhibited three pi-cation interactions through involvement of Lys407 (thiazole, 4.23 Å and 4-fluoro phenyl amine of 1,3,5-triazine, 4.38 Å) and Lys483. The compound **14** formed two hydrogen bonds with Lys407 and Ser476 through nitrogen of 1,3,5-triazine and amine bridge connecting 4-chloro phenyl amine to 1,3,5-triazine (2.36 Å and 2.56 Å, respectively). On the other hand, it also displayed two pi-cation interactions with Lys407 (with thiazole, 4.24 Å and 4-chloro phenyl amine connected to 1,3,5-triazine, 3.50 Å) and Lys483 through 1,3,5-triazine with interatomic distance of 3.73 Å. No considerable change in hydrogen bond and pi-cation interaction was observed on replacing 4-chloro with 4-bromo except one hydrogen bond through nitrogen of 1,3,5-triazine with Tyr487 (2.44 Å) as in the case of compound **15**. However, it displayed two pi-cation interactions by means of Lys407 (3.50 A and 5.18 Å) and Lys483 (3.33 Å).

From post-docked posses of ligands obtained from docking, it was inferred that molecules were deeply buried and stabilised into the active site of *candida albicans* cytosolic leucyl-tRNA synthetase editing domain through prolific intriguing attractions by hydrogen bonds and pi-cation interactions. For majority of molecules, Lys483, Lys407 and Asp422 were identified as key protein residues responsible for generation of hydrogen bonds and Asp421, Leu317, Arg318, Ser476, Ala315 and Tyr487 for minor interaction. Whereas, for pi-cation interactions, it were observed that Lys407 and Lys483 were the only responsible residues. Amine bridge, thiazole, 1,3,5-triazine, substituted phenyl connected to thiazole and 1,3,5-triazine were worked out as major structural features imperative for interactions.

### Scoring

Piecewise Linear Potential (PLP) is a fast and simple docking function that has been shown to correlate well the structural features with protein ligand binding affinities. Higher PLP scores indicate stronger receptor-ligand binding. Two versions of the PLP function are available: PLP1 and PLP2. In order to better define binding affinity of ligands with receptor, post-docked ligand-receptor pose was evaluated on the basis of scoring parameters like PLP1, PLP2, PMF, ligand internal energy and dock score. These molecules were reported to show considerable binding affinity for the receptors as shown by PLP scores ranging from 42.84 (compound **9**) to 106.93 (compound **14**). The PMF scoring functions were developed based on statistical analysis of the 3D structures of protein-ligand complexes. These were found to correlate well with protein-ligand binding free energies while being fast and simple to calculate. As depicted in the Table 2, molecules having halogen substituted phenyl amine connected to 1,3,5-triazine nucleus, showed higher PMF values than their counterparts. The internal nonbonded ligand energy is also calculated for each new conformation generated. It consists of a van der Waals (vdW) term and an optional electrostatic term. All molecules disclosed optimum energy level, however, the Compound **7** (-10.55) and **8** (-10.20) were more stabilised. Lastly, candidate ligand poses were evaluated and prioritized according to the dock score function and the entire dataset revealed extensive score value ranging from 50.58 (**9**) to 75.68 (**14**).

## Conclusion

In conclusion, hybrid phenylthiazole-1,3,5-traizine derivatives may act as *candida albicans* cytosolic leucyl-tRNA synthetase inhibitors on the basis of molecular docking runs which contribute to the possible mechanism of antifungal activity of these analogues. These molecules were energetically proficient enough to make stable contacts with target protein on account of effective hydrogen bond and pi-cation interactions, which was also supplemented through scoring parameters.

In the light of above observation, the conjugates of phenylthiazole and 1,3,5-triazine can act as a lead towards the development of potential leucyl-tRNA synthetase inhibitors, albeit, a considerable amount of work is still required in this direction and it is in progress in our laboratory and will be reported subsequently in future.
